# The role of amputation as an outcome measure in cellular therapy for critical limb ischemia: implications for clinical trial design

**DOI:** 10.1186/1479-5876-9-165

**Published:** 2011-09-27

**Authors:** Eric Benoit, Thomas F O'Donnell, Mark D Iafrati, Enrico Asher, Dennis F Bandyk, John W Hallett, Alan B Lumsden, Gregory J Pearl, Sean P Roddy, Krishnaswami Vijayaraghavan, Amit N Patel

**Affiliations:** 1 The CardioVascular Center, Tufts Medical Center, Boston, MA 02111, USA; 2 Division of Vascular Surgery, Maimonides Medical Center, Brooklyn, NY 11219, USA; 3 Division of Vascular & Endovascular Surgery, University of Southern Florida, Tampa, FL 33606, USA; 4 Heart & Vascular Center, Roper St. Francis Medical Center, Charleston, SC 29401, USA; 5 Methodist DeBakey Heart & Vascular Center, The Methodist Hospital, Houston, TX 77030, USA; 6 Department of Vascular Surgery, Baylor University Medical Center, Dallas, TX 75204, USA; 7 The Vascular Group, Albany, NY 12208, USA; 8 Department of Vascular Surgery, Sri Ramachandra Medical College & Research Institute, Chennai, India; 9 Division of Surgery, University of Utah, Salt Lake City, UT 84132, USA

## Abstract

**Background:**

Autologous bone marrow-derived stem cells have been ascribed an important therapeutic role in No-Option Critical limb Ischemia (NO-CLI). One primary endpoint for evaluating NO-CLI therapy is major amputation (AMP), which is usually combined with mortality for AMP-free survival (AFS). Only a trial which is double blinded can eliminate physician and patient bias as to the timing and reason for AMP. We examined factors influencing AMP in a prospective double-blinded pilot RCT (2:1 therapy to control) of 48 patients treated with site of service obtained bone marrow cells (BMAC) as well as a systematic review of the literature.

**Methods:**

Cells were injected intramuscularly in the CLI limbs as either BMAC or placebo (peripheral blood). Six month AMP rates were compared between the two arms. Both patient and treating team were blinded of the assignment in follow-up examinations. A search of the literature identified 9 NO-CLI trials, the control arms of which were used to determine 6 month AMP rates and the influence of tissue loss.

**Results:**

Fifteen amputations occurred during the 6 month period, 86.7% of these during the first 4 months. One amputation occurred in a Rutherford 4 patient. The difference in amputation rate between patients with rest pain (5.6%) and those with tissue loss (46.7%), irrespective of treatment group, was significant (p = 0.0029). In patients with tissue loss, treatment with BMAC demonstrated a lower amputation rate than placebo (39.1% vs. 71.4%, p = 0.1337). The Kaplan-Meier time to amputation was longer in the BMAC group than in the placebo group (p = 0.067). Projecting these results to a pivotal trial, a bootstrap simulation model showed significant difference in AFS between BMAC and placebo with a power of 95% for a sample size of 210 patients. Meta-analysis of the literature confirmed a difference in amputation rate between patients with tissue loss and rest pain.

**Conclusions:**

BMAC shows promise in improving AMP-free survival if the trends in this pilot study are validated in a larger pivotal trial. The difference in amp rate between Rutherford 4 & 5 patients suggests that these patients should be stratified in future RCTs.

## Background

Over the last decade there have been major changes in the therapeutic approach to peripheral arterial occlusive disease (PAD) resulting in improved outcomes and a decrease in overall mortality [1]. In a review of an administrative database (Medicare part B claims) Goodney and associates showed a threefold increase in endovascular procedures and a dramatic decrease of 42% in bypass surgery [2]. This shift to less invasive therapy was accompanied by a significant 25% decrease in major lower extremity amputations. Despite this progress there are a considerable number of critical limb ischemia patients (CLI), who have exhausted their therapeutic options- the so called no option CLI group (NOCLI). Various innovative approaches have been employed in these patients who are beyond the typical catheter-based or open surgical techniques. Unfortunately, in randomized controlled trials (RCTs) spinal cord stimulation and prostaglandin infusion have not led consistently to an improved clinical state. Autologous cellular therapy, however, has been associated with promising results, but has not undergone extensive and rigorous testing by randomized control trials.

TASC I/II suggested that the combined endpoint of amputation-free survival (AFS) was the best outcome measure to assess patients with CLI [3]. The hallmark BASIL trial which randomized CLI patients between angioplasty an open bypass surgery employed AFS as its primary outcome [4]. Despite the wishes of investigators to employ different primary endpoints such as improvement in: pain, the clinical status of the limb (Rutherford score), disease specific Quality of Life (QoL), and the various surrogate measures of Ankle Brachial Index (ABI) and Transcutaneous pressure of Oxygen (TCpO2), AFS has been recommended as the gold standard. Of the many efficacy outcomes with CLI there has been little detail from controlled trials on the ultimate goal of the therapy -- the prevention of major amputation (AMP), which is combined with mortality over time to provide AMP-free survival (AFS). Except for overwhelming sepsis the decision and timing for AMP is both physician and patient driven. Only a trial which is double blinded for both the treating physician and the patient can eliminate bias as to the timing and reason for AMP.

The purpose of this study is to describe the 6 month outcomes for a randomized controlled trial of autologous bone marrow derived stem cells for no option CLI patients. The focus of this report will be on the description of the timing of, number of and reason for amputations in both the treatment and placebo groups from a pilot trial approved by the FDA. The purpose of this trial was to examine safety features of bone marrow derived cells implanted intramuscularly in the limbs of no-option CLI patients. The control group also underwent iliac crest puncture, but not withdrawal of cells. The placebo group received peripheral blood as the injectate. Since this was a safety study, it was not powered for efficacy. The safety data, three month data, and a detailed description of the protocol has been presented previously [5]. Based on these findings and a review of the literature the implications for designing a pivotal trial in no option CLI will be discussed.

## Methods

A detailed description of the randomized, double-blinded controlled trial has been presented previously [5].

### Patients

Patients qualified for inclusion if they had chronic, critical limb ischemia including rest pain (Rutherford class 4) or mild-to-moderate tissue loss (Rutherford 5) and were not candidates for surgical or endovascular revascularization. Hemodynamic parameters included one of the following: ankle pressure < 50 mmHg or ABI < 0.4; toe pressure < 40 mmHg or TBI < 0.4; or TcPO2 < 20 mmHg on room air.

Exclusion criteria included extensive necrosis of the index limb making amputation inevitable (Rutherford class 6); uncorrected iliac artery occlusion ipsilateral to index limb; lack of Doppler signal in the index limb (ABI = 0); serum creatinine ≥ 2.0 mg/dL; active infection requiring antibiotics; active malignancy; or any hematologic disorder that prevented bone marrow harvesting.

All patients were ≥ 18 years of age and able to provide informed consent. All enrolled patients underwent pre-operative cancer screening and ophthalmologic examinations for proliferative retinopathy.

### Marrow aspiration, processing & injection

Patients randomized to the cell therapy group underwent aspiration of 240 mL of bone marrow from the iliac crest, and aspirate was then processed into 40 mL of concentrate using the SmartPReP^®^2 Bone Marrow Aspirate Concentrate (BMAC) system (Harvest Technologies, Plymouth, Massachusetts). This automated, point-of-care centrifuge system has been previously used for autologous cell therapy in CLI [6,7]. At the time of IV placement 10 mL of peripheral blood was withdrawn from each patient. Patients randomized to the control group had this blood diluted 3:1 and presented in a syringe for injection. The vascular surgeon made 40 intramuscular injections of 1 mL aliquots of either BMAC (experimental group) or dilute peripheral blood (control group) into previously identified locations along the ischemic limb using ultrasound guidance. Procedures were carried out under local anesthesia and conscious sedation.

### Randomization & blinding

Because the decision to amputate is patient and physician driven, it may be biased by knowledge of treatment group; therefore, allocation concealment and blinding of both patient and treating vascular surgeon were approached very meticulously in this study. Patients were centrally randomized to investigational treatment or placebo in a 2:1 ratio. 48 patients were enrolled with 34 receiving cell therapy and 14 receiving placebo injections. Study group assignment was revealed in the operating room after prepping and draping but prior to marrow harvesting. Subject randomization was revealed only to the individual performing the bone marrow aspiration. To maintain blinding of patients, bilateral iliac punctures were performed on all subjects. Treatment patients had 240 mL marrow aspirated whereas control patients had 2 mL marrow aspirated. Following centrifugation of the aspirate (treatment group) or sham operation of the centrifuge (control group), the unblinded physician and study coordinator left the procedure room and the blinded vascular surgeon and blinded study coordinator entered. The surgeon was presented with four syringes for injection without knowing its contents. For treatment patients this was 40 mL of BMAC; for control patients it was 40 mL of diluted peripheral blood. Effectiveness of blinding was assessed by querying the patients and clinicians after the procedure and at the conclusion of the study.

### Follow up & Outcome measurements

Patients were evaluated at 1, 4, 8, 12 and 26 weeks post-procedure. Clinical outcomes included amputation status, Rutherford classification of limb ischemia, and pain as determined by Visual Analog Scale (VAS). Major amputations were defined as those occurring above the ankle. Hemodynamic outcome was evaluated by Ankle Brachial Index (ABI). Laboratory monitoring of hematology and blood chemistries was also performed. Ophthalmologic retinal examination was performed at baseline and 12 weeks in diabetics to evaluate for proliferative retinopathy.

### Literature review

To define a baseline amputation rate in No Option CLI, we reviewed the literature for clinical trials of No Option CLI patients and identified the control groups as a surrogate natural history population. We queried the PubMed and the Cochrane database using terms such as "critical limb ischemia," "amputation," "randomized controlled trial," and non-revascularization treatments such as "cell therapy," "spinal cord stimulation," "gene therapy," "prostaglandin therapy." We included only clinical trials published in the English literature within the last 15 years, excluding case reports and case series of fewer than 5 patients. We excluded papers concerning acute limb ischemia, surgical or endovascular revascularization, ischemia due to diseases other than atherosclerosis (such as thromboangiitis obliterans), and trials where the CLI portion of the patient population could be separately evaluated.

### Statistical modeling

A bootstrap simulation approach was used to model results for a future pivotal trial. This involved using data from the pilot trial as input, modifying certain variables, and performing simulated trials multiple times to quantify the odds of obtaining significant results in a larger trial.

## Results

### Patients & Procedure

Patient baseline characteristics are presented in Table 1. All patients had atherosclerotic arterial occlusive disease. It is notable that the treatment group contained a slightly greater proportion of patients with tissue loss (Rutherford 5) than did the control group (67.6% vs 50%) although this difference was not statistically significant. All marrow aspiration, processing and limb injection was accomplished in the operating room at a single visit. To determine the effectiveness of blinding, patients and investigators were asked prior to discharge following treatment to identify which treatment arm the subject had been assigned. Collected responses were used to determine a blinding index which demonstrated successful blinding of both patients and investigators, as reported previously [5].

**Table 1:** Patient demographics

	BMAC	Control	Total
Patients	34	14	48

Age (years)	72.5	65.7	69.5
	(42 - 93)	(52 - 85)	(42 - 93)

Male (n)	23	9	32
	(7.6%)	(64.3%)	(66.7%)

Diabetes Mellitus			
Type 1	1	1	2
	(2.9%)	(7.1%)	(4.2%)
	
Type 2	17	5	22
	(50.0%)	(35.7%)	(45.8%)
	
Type 1 + Type 2	18	6	24
	(52.9%)	(42.9%)	(50.0%)

Renal insufficiency (Cr clearance< 40 mg/dL)	3	1	4
	(8.8%)	(7.1%)	(8.3%)

Cardiac disease			
Angina	4	3	7
	(11.8%)	(21.4%)	(14.6%)
	
Myocardial infarction	11	2	13
	(32.4%)	(14.3%)	(27.1%)
	
Congestive heart failure	7	4	11
	(20.6%)	(28.6%)	(22.9%)
	
Other cardiac	20	11	31
	(58.8%)	(78.6%)	(64.6%)

Coronary revascularization			
Coronary artery bypass	11	2	13
	(32.4%)	(14.3%)	(27.1%)
	
Coronary angioplasty	5	6	11
	(14.7%)	(42.9%)	(22.9%)

Rutherford class			
Rutherford 4	11	7	18
	(32.4%)	(50.0%)	(37.5%)
	
Rutherford 5	23	7	30
	(67.6%)	(50.0%)	(62.5%)

Ankle brachial index (ABI)	0.46	0.396	0.44
	(0.13-1.23)	(0.17-0.74)	(0.13-1.23)

Previous amputation (contralateral)			
Major amputation	7	1	8
	(20.6%)	(7.1%)	(16.7%)
	
Minor amputation	6	2	8
	(17.6%)	(14.3%)	(16.7%)

Previous lower extremity revascularization			
Surgical bypass	23	8	31
	(67.6%)	(57.1%)	(64.6%)
	
Angioplasty	8	7	15
	(23.5%)	(50.0%)	(31.3%)
	
Stent	5	6	11
	(14.7%)	(42.9%)	(22.9%)
	
Atherectomy	2	4	6
	(5.9%)	(28.6%)	(12.5%)

Reason for no option status			
Failed revascularization	18	6	24
	(52.9%)	(42.9%)	(50.0%)
	
Anatomic poor candidate	31	12	43
	(91.2%)	(85.7%)	(89.6%)
	
Medical high risk	5	1	6
	(14.7%)	(7.1%)	(12.5%)
	
Other	1	1	2
	(2.9%)	(7.1%)	(4.2%)

Medication			
Cholesterol lowering agent	24	11	35
	(70.6%)	(78.6%)	(72.9%)
	
Beta blocker	15	5	20
	(44.1%)	(35.7%)	41.7%)
	
Antiplatelet agent	28	14	42
	(82.4%)	(100.0%)	(87.5%)

### Safety & Adverse Events

This data was presented previously and will be briefly summarized here [5]. Bone marrow aspiration was well tolerated. Study patients experienced a mean decrease in hematocrit 2.6 percent compared with controls. However this drop was transient, associated with no hemodynamic instability, and required no specific therapy in any study patients. Injection of ischemic limbs was associated with no evidence of muscle damage, either clinically or by elevation in creatine phosphokinase. No patient developed acute kidney injury. No patient developed inappropriate angiogenesis such as vascular malformations or arteriovenous fistulae in the index limb or remote sites. Four patients demonstrated proliferative retinopathy at screening, but there were no cases of new or worsened retinopathy at follow up. There were no cases of new or recurrent malignancy identified during follow up.

### Median follow-up

The median follow up for the 48 patients in this series was 346 days (range 8 - 783 days). This report will focus on the six month data. No patients were lost to follow up.

### Amputation 6 months

Fifteen amputations occurred within the six month follow up period. One late amputation was performed at 388 days into the trial and was not included in the six month analysis. At six months, the 10 of 34 patients in the BMAC group had undergone amputation (29.4%) while the control group had 5 of 14 patients amputated. (35.7%) (p = 0.412). (Table 2) When evaluating the amputation rate by Rutherford classification in all patients, the Rutherford 4 group had 1 amputation, while the Rutherford 5 group had 14, irrespective of treatment group, (5.6% vs 46.7%, p = 0.0029). During the six month period 93.3% of all amputations performed occurred in the Rutherford 5 group. Within the Rutherford 5 group, 9 of 23 BMAC patients (39.1%) underwent amputation as compared to 5 of 7 control patients (71.4%) (p = 0.1337). (Table 3). Of the 15 amputations, 86.7% occurred within 4 months.

**Table 2:** Six month amputation rate by Rutherford class

	AMP	Tot	% AMP	p *
All Pts	15	48	31.3%	
R4	1	18	5.6%	0.0029
R5	14	30	46.7%	

BMAC	10	34	29.4%	
R4	1	11	9.1%	0.0721
R5	9	23	39.1%	

Control	5	14	35.7%	
R4	0	7	0.0%	0.0053
R5	5	7	71.4%	

* p calculated using Fischer's exact test

**Table 3:** Six month amputation rate: BMAC vs Control, Rutherford 5 only

R5	AMP	Tot	% AMP	p *
BMAC	9	23	39.1%	0.1337
Control	5	7	71.4%	

* p calculated using Fischer's exact test

### Indications for amputation

Table 4 presents the indications for amputation. Most patients presented with multiple indications. Pain was an indication in 13 of 15 amputations, and 7 of these patients scored a maximum 100 on the VAS pain scale.

**Table 4:** Indications for amputation

	N	% Amps
Pain	13	86.67%
Gangrene	6	40.00%
Disease progression	5	33.33%
Local infection	5	33.33%
Systemic infection	1	6.67%
Failure to heal	1	6.67%
Osteomyelitis	0	0.00%

### Six month mortality

There were two deaths during the 6 month period. One BMAC patient died. One control patient died following an amputation and was therefore censored from analysis of AFS.

### Time to amputation and Amputation free survival

**Figure 1:**
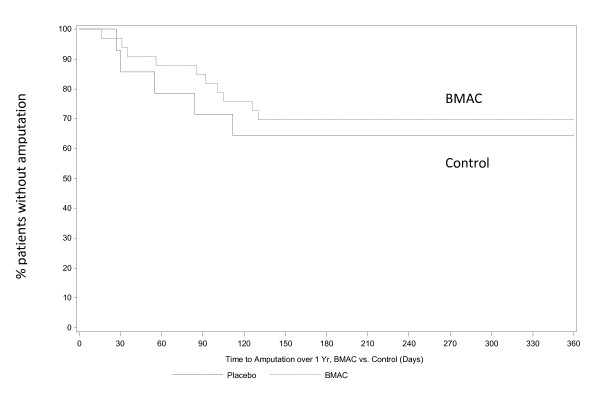
**Time to Amputation over 1 year BMAC vs. Control (All study patients)**. The Kaplan-Meier curve demonstrates the time to amputation over 1 year between BMAC and control patients in all study subjects.

**Figure 2:**
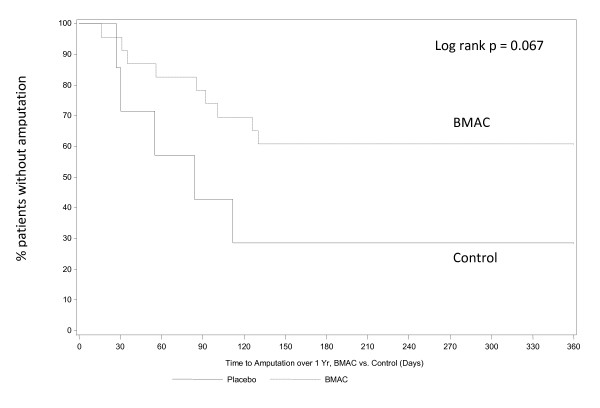
**Time to Amputation over 1 year BMAC vs. Control (Rutherford 5 patients only)**. There were no amputations in the Rutherford 4 group, removing these patients results in separation of the BMAC and control curves and demonstrates a trend towards a positive treatment effect on amputation. (p = 0.067)

**Figures 3:**
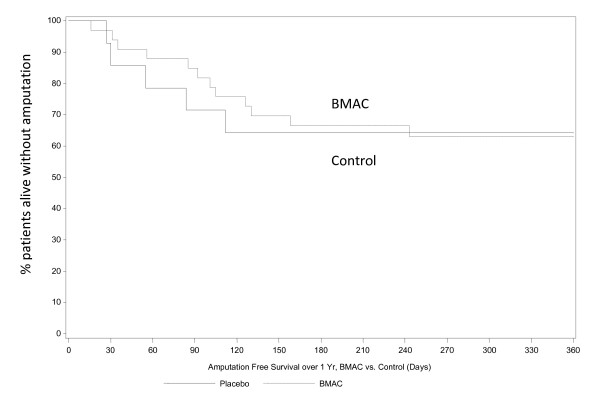
**Amputation Free Survival over 1 year BMAC vs. Control (All study patients)**. The Kaplan-Meier curve demonstrates Amputation Free Survival (AFS) over 1 year in all study subjects with a negligible difference between BMAC and control.

**Figure 4:**
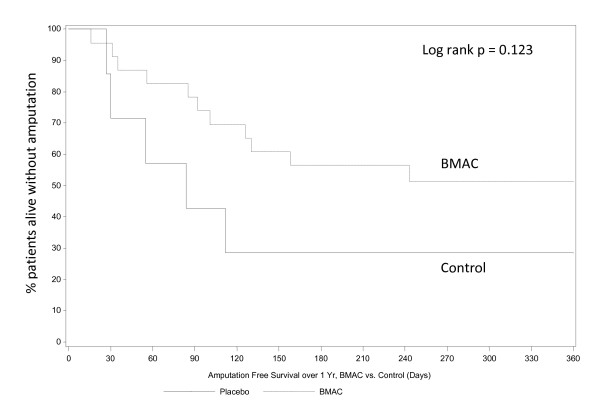
**Amputation Free Survival over 1 year BMAC vs. Control (Rutherford 5 patients only) displays**. The separation of AFS curves demonstrates a tread towards a positive effect of BMAC on AFS (p = 0.123). As in Figure 1, the event rate in Rutherford 4 patients was low, diluting any apparent treatment effect.

The median time to amputation was 88.5 days in the BMAC group and 55.0 days in the control group (p = 0.358). Time to amputation is presented as Kaplan-Meier curves in Figures 1 &2 comparing the impact of BMAC treatment on all patients (Figure 1) and just Rutherford 5 patients (Figure 2). Kaplan-Meier curves for amputation free survival in all subjects (Figure 3) and Rutherford 5 patients only (Figure 4). Looking at patients with tissue loss only, the separation of treatment and control curves is more apparent, demonstrating a modest treatment effect. For amputation, the Log rank p value is 0.067, for AFS the Log rank p value is 0.123. Patients with tissue loss had a lower AFS than those without (50.0% vs 94.4%, p = 0.0016)

### Regression analysis of patient factors on Amputation rate & AFS

Using a logistic regression model, we analyzed the effect of diabetes, age > 75 years, and tissue loss on amputation rate and AFS. While diabetes and age were not found to correlate with either outcome, tissue loss had a statistically significant correlation with both amputation at 6 months (Odds Ratio 18.48, p = 0.0103) and amputation free survival (OR 20.15, p = 0.0078).

### Change in Rutherford Class

We evaluated change in Rutherford class using a binary approach. Patients who improved at least one numeric category (e.g., 5 → 4) were defined as improved. Patients who failed to improve or deteriorated at least one class were defined as worse; amputation was scored as deterioration of Rutherford Class: stable or worsening Rutherford class. (See Table 5) The overall rate of improvement was 35.4%, but when evaluated by baseline category, the difference in improvement rates was statistically significant. For all patients, 66.7% of Rutherford 4 patients improved while only 16.7% of Rutherford 5 patients did (p = 0.0005). In the BMAC group, more Rutherford 4s improved than Rutherford 5s (81.8% vs 17.4%, p = 0.0003) Looking at the effect of treatment on Rutherford 4 patients alone, 81.8% of the BMAC patients improved while 42.9% of control patients improved (p = 0.0874).

**Table 5:** Change in Rutherford class at 6 months

	Improved	Worse	% Imp	Total	p *
Total	17	31	35.4%	48	
R4	12	6	66.7%	18	0.0005
R5	5	25	16.7%	30	

BMAC	13	21	38.2%	34	
R4	9	2	81.8%	11	0.003
R5	4	19	17.4%	23	

Control	4	10	28.6%	14	
R4	3	4	42.9%	7	0.2367
R5	1	6	14.3%	7	

* p calculated using Fischer's exact test

### Change in ABI

Mean change in ABI by treatment group was not significant and such analysis is hampered by patients lost to amputation and missing data points We therefore evaluated ABI in a binary fashion with improvement defined as increase in ABI ≥ 0.1 per TASCII. Failure to improve was defined as failure to increase ABI by at least 0.1, decrease in ABI, amputation or death. Missing data was also counted as failure to improve. While overall Rutherford 4 patients demonstrated a greater degree of improvement than Rutherford 5 patients, this was not significant. There was no difference between treatment and control. This analysis was complicated by multiple missing data points.

### Effect of tissue loss on power calculations

The event rate in Rutherford 4 patients is significantly less than that in Rutherford 5 patients. We conducted a sensitivity analysis to determine the impact of increasing proportions of Rutherford 4 patients on the power calculations to identify a treatment effect on amputation rate in a pivotal study.

### Bootstrap simulation

A bootstrap approach was used to simulate data 1,000 times, using the event rate of AFS component events (AMP + mortality) in the pilot trial. Given the low amputation rate in rest pain patients, we limited the simulation to Rutherford 5 patients (BMAC 43.5%, control 71.4%). A Cox proportional hazard model was used for analysis of 140 BMAC treated patients and 70 placebo treated patients. Using this approach 974 out of 1,000 simulations resulted in a statistically significant treatment effect (p < 0.05). The results indicate a 97.4% chance of showing a statistically significant treatment effect extrapolating from pilot data in patients with tissue loss.

### Amputation rate in the No Option CLI literature

We identified 8 studies in the literature that presented 6 month amputation rates in NOCLI patients. One trial was a retrospective review of 5 years of unreconstructable CLI patients at an academic medical center with 1 year follow up [8]. The remaining papers were randomized, controlled trials: three spinal cord stimulation studies [9-11]; one pharmacologic trial of a prostaglandin E1 analog (CIRCULASE) [12]; two gene therapy trials of NV1FGF (TALISMAN [13] and TAMARIS [14]; and one cell therapy trial [15]. To this group we added the data from the current BMAC cell therapy trial. Using only the control groups from these trials, we identified an overall six month amputation rate of 23.0% in this surrogate natural history population of NOCLI. (Table 6)

**Table 6:** Six month amputation in NO-CLI Control groups only

	N	Amp N	Amp %
Jivegard 1995	26	10	38.5%
Lepantalo 1996	136	53	39.0%
Klomp 1999	60	20	33.3%
Amann 2003	39	15	38.5%
Brass 2006	190	16	8.4%
Nikol 2008	56	16	28.6%
Hiatt 2010	259	44	17.0%
Powell 2011	24	6	25.0%
Benoit 2011	14	5	35.7%

	804	185	23.0%

NO-CLI: No option critical limb ischemia

To evaluate the impact of tissue loss on 6 month amputation rate we performed a meta-analysis of three studies that compared amputation rate between patients with rest pain and those with tissue loss [10,12]. These trials comprised 264 patients, 114 with rest pain and 150 with tissue loss. Taken together, these trials demonstrate that tissue loss correlates with an increased amputation risk. (Table 7)

**Table 7:** Six month amputation rates by Rutherford class

Rest pain/R4	N	Amp N	Amp %
Brass 2006	88	3	3.4%
Klomp 1999	19	3	15.8%
Benoit 2011	7	0	0.0%

	114	6	5.3%
			
**Tissue Loss/R5**	**N**	**Amp N**	**Amp %**

Brass 2006	102	12	11.8%
Klomp 1999	41	16	39.0%
Benoit 2011	7	5	71.4%

	150	33	22.0%

## Discussion

This randomized, double-blinded, controlled trial of autologous stem cell therapy for critical limb ischemia was a pilot trial and intended to present a safety profile for the technique and to identify trial design issues for a pivotal trial. Although not powered to demonstrate efficacy, the outcomes provide valuable trends about future trial design, not only for this technique, but for other trials in CLI.

There was a trend toward improved amputation rates in Rutherford 5 patients treated with BMAC, although this did not achieve statistical significance. (p = 0.1337) More important was the difference in amputation rates between those patients with rest pain at screening (Rutherford 4) and those with tissue loss (Rutherford 5). Patients with tissue loss demonstrated a much higher amputation event rate (46.7%) than did those with rest pain alone (5.6%) and this difference was statistically significant (p = 0.0029). Only a single patient with rest pain underwent an amputation by six months; patients with tissue loss at screening accounted for 93.3% of all amputations. This association was further confirmed by logistic regression modeling which demonstrated the correlation of tissue loss with amputation with an Odds Ratio of 18.48 (p = 0.013).

With regard to change in Rutherford class as an instrument to measure effectiveness of CLI therapy, we found the opposite. A higher percentage of Rutherford 4 patients demonstrated an improvement in Rutherford classification than did Rutherford 5 patients. This makes biologic sense, because an ischemic ulcer requires more blood flow to heal than intact skin does to survive. Therefore salvaging a limb with tissue loss requires a far greater degree of perfusion than does achieving resolution of rest pain [3]. While change in Rutherford class may be a used to evaluate therapy in patients with rest pain, it may not be as appropriate for advanced CLI patients.

ABI was a difficult endpoint to evaluate because of multiple missing data points and the difficultly of incorporating amputation into this measure. Hemodynamic endpoints may not be appropriate for NOCLI patients at high risk for amputation.

Since the results in our pilot trial differed depending on the Rutherford classification of patients at screening, we surveyed the literature for evidence of the impact of tissue loss on the amputation rate. To determine the amputation rate in no option CLI patients overall, we identified a group of NOCLI patients gathered from the control groups of eight randomized trials and one observational study in the literature. This surrogate natural history population demonstrated a six month amputation rate of 23%. However, only the CIRCULASE trial, Klomp's spinal cord stimulation trial and the current BMAC pilot trial present amputation data according to Rutherford class. Meta-analysis of these trials demonstrated that patients with tissue loss have a higher amputation rate than those with rest pain (Hazard Ratio 8.650, p = 0.0513). This is further supported by a separate analysis of patients in a spinal cord trial in which those with tissue loss demonstrated a higher amputation risk (HR 2.38, p = 0.018) [16].

Because most published clinical trials group all CLI patients together regardless of disease severity, it is difficult to quantify the impact of tissue loss on amputation rate. However, there is evidence to support that tissue loss in CLI correlates with poor outcomes. In addition to an analysis of NOCLI studies, risk stratification models underline the correlation between tissue loss and amputation in patients undergoing surgical or endovascular revascularization. The first of two validated risk stratification models, the PREVENT III tool was derived from a multivariate analysis of nearly a thousand CLI patients, and it identified tissue loss as second only to dialysis as a factor negatively impacting AFS. In a population of patients undergoing surgical bypass, tissue loss was associated with a hazard ratio of 2.2 for increased risk of death or amputation at one year [17]. The Finnvasc model identified tissue loss as a predictor of negative outcome during the immediate postoperative period [18]. Worse scores on either of these tools correlate with decreased AFS at one year in patients undergoing revascularization [19].

Tissue loss correlates with a variety of poor outcomes in CLI. Khan et al. demonstrated that in patients undergoing surgical revascularization, tissue loss is associated with a higher rate of amputation even if the revascularized segment remained patent - the so-called anatomic success but functional failure [20]. Nguyen et al. found that in patients undergoing surgical bypass those with tissue loss required increased resource utilization such as length of stay and experienced a higher rate of graft related events (GRE) such as thrombosis, need for revision or amputation [21]. In a prospective observational study of 1560 CLI patients, of whom 36.5% underwent revascularization, Bertele et al. found that tissue loss was associated with increased risk of amputation at six months (7.8% vs. 13.9%) [22]. Taylor et al. reviewed 2240 limb revascularizations for PAD according to preoperative indication [23]. The population included patients with claudication (999), ischemic rest pain (464), and tissue loss (777). There were significant differences in multiple endpoints, including limb salvage and survival, among the groups. The 1 year limb salvage rate for patients with rest pain was 88.6% while for those with tissue loss it was 75.1% (p < 0.001). The survival rate at one year was 79.1% for rest pain and 65.8% for tissue loss (p < 0.001).

### Implications for trial design

This difference in outcomes between patients with tissue loss and those with rest pain has implications for study design in CLI. Patients with tissue loss demonstrate a higher rate of amputation, which is a component of amputation free survival (AFS). If amputation rate or AFS are used as the primary endpoint in a clinical trial, the lower number of amputations in Rutherford 4 patients may dilute the event rate in the overall patient population making it difficult to demonstrate a treatment effect. This is supported by sensitivity analysis which demonstrates that increasing the proportion of Rutherford 4 patients in the study population requires increasingly larger sample sizes to maintain appropriate statistical power. Patients with tissue loss are appropriately evaluated using amputation or AFS as an endpoint.

On the other hand, if the outcome measure is change in Rutherford class, which has been suggested by some as component of a combined endpoint, a sample weighted towards Rutherford 4 may better demonstrate changes by this measure.

Recently some authors have begun to question whether grouping all CLI patients together obscures the evaluation of therapy [23-25] and our findings confirm that patients with tissue loss should be analyzed separately from those with rest pain.

## Conclusions

Our experience with a pilot trial of cell therapy and a review of the literature suggest that CLI patients with rest pain behave differently than those with tissue loss. If amputation or amputation free survival are to be used as primary endpoints for CLI trials, study subjects should be stratified by Rutherford class or limited only to Rutherford 5 patients in order to best demonstrate a treatment effect of therapy.

## Competing interests

Drs. Benoit and O'Donnell have served as medical consultants for Harvest Technologies, Inc. (Plymouth, Massachusetts). Dr. Patel has other research funding for clinical trials from Harvest Technologies, Inc. (Plymouth, Massachusetts).

## Authors' contributions

EB, TO, MI, and AP where all equally involved in creation and implementation of the clinical trials and writing of the manuscript. EA, MI, DB, JH, AL, GP, SR, KV, and AP were all equally involved with enrolling patients and manuscript editing.

All authors read and approved the final manuscript.
